# Feasibility Assessment of Optical Coherence Tomography-Guided Laser Labeling in Middle Cranial Fossa Approach

**Published:** 2018-11

**Authors:** Saleh Mohebbi, Jakob Lexow, Alexander Fuchs, Thomas Rau, Sebastian Tauscher, Marjan Mirsalehi, Seyed Mousa Sadr Hosseini, Tobias Ortmaier, Thomas Lenarz, Omid Majdani

**Affiliations:** 1 *Brain and Spinal cord Injury Research Center, Neuroscience Institute,* *Tehran University of Medical Science, Tehran, Iran.*; 2 *Department of Otorhinolaryngology, Hannover Medical School, Hannover, Germany. *; 3 *Institute of Mechatronic Systems (IMES), Leibniz Universität Hannover, Hannover, Germany.*; 4 *ENT Head and Neck Research Center and Department, Rasool Akram Hospital, Iran University of Medical Science, Tehran, Iran. *

**Keywords:** Computer-assisted surgery, Er-YAG laser, Image-guided surgery, Middle cranial fossa, Optical coherence tomography

## Abstract

**Introduction:**

Different approaches have been developed to find the position of the internal auditory canal (IAC) in middle cranial fossa approach. A feasibility study was performed to investigate the combination of cone beam computed tomography (CBCT), optical coherence tomography (OCT), and laser ablation to assist a surgeon in a middle cranial fossa approach by outlining the internal auditory canal (IAC).

**Materials and Methods:**

A combined OCT laser setup was used to outline the position of IAC on the surface of the petrous bone in cadaveric semi-heads. The position of the hidden structures, such as IAC, was determined in MATLAB software using an intraoperative CBCT scan. Four titanium spheres attached to the edge of the craniotomy served as reference markers visible in both CBCT and OCT images in order to transfer the plan to the patient. The integrated erbium-doped yttrium aluminum garnet laser was used to mark the surface of the bone by shallow ablation under OCT-based navigation before the surgeon continued the operation.

**Result:**

The technical setup was feasible, and the laser marking of the border of the IAC was performed with an overall accuracy of 300 μm. The depth of each ablation phase was 300 μm. The marks indicating a safe path supported the surgeon in the surgery.

**Conclusion:**

The technique investigated in the present study could decrease the surgical risks for the mentioned structures and improve the pace and precision of operation.

## Introduction

Different tumors arise in the temporal bone, such as paraganglioma, meningioma, and schwannoma of the cranial nerves. The internal auditory canal (IAC) is also a focus of interest due to being located in the vicinity of prevalent tumors (e.g., vestibular schwannoma) and other cranial nerves (e.g., the facial and cochlear nerves). Skull base fracture may also involve the temporal bone and lead to hearing loss or facial paralysis.

There are several surgical approaches to access the IAC.Retrosigmoid, translabyrinthine, sub-temporal, or middle cranial fossa (MCF) approaches are the main methods for tumor removal. In this regard, MCF is a good choice for small tumors with a minimal cisternal part that facilitates the access to IAC with or without a minimal hearing disturbance. Facial nerve decompression or exposure is another surgical indication for this approach. In this method, the surgeon has to work in a narrow and small space and with a angled view. 

There are several important structures present in the surface of the petrous bone, such as the facial nerve, geniculate ganglion, superior semicircular canal, and cochlea (1). The margins of the IAC are not defined at the superior surface of the temporal bone; therefore, it is not clearly visible as an anatomically marked structure to the surgeon during the dissection. Another important point is the surgical time because of the brain retraction in this procedure.

Image-guided surgery (IGS) is still not popular in lateral skull base. The main reason for this is the inaccuracy of commercially available IGS system. The lateral skull base surgery requires a sub-millimeter accuracy. In addition, most of the currently available IGS systems are using pre-operative image data and not updating the baseline imaging in the real-time.Optical coherence tomography (OCT) is a high-resolution imaging system that works fundamentally through the light. Huang et al. first described medical OCT (2). The OCT is gaining interest due to its very high resolution (3). The use of OCT can increase the accuracy of IGS. This non-contact technology requires no sample preparation and presents no radiation hazards (2,3). Laser is a good choice for precise labeling on the surface of a bone. Carbon dioxide laser (CO_2 _laser) and erbium-doped yttrium aluminum garnet laser (Er:YAG laser) have demonstrated effectiveness for bone tissue ablation, cutting, or marking (4,5,6). However, precisely guiding the ablation without harming neighboring structures requires a highly accurate navigation. One of the best candidates for this task is OCT (7). There are a number of studies examining the combination of OCT and a laser system in one machine and evaluating the adapted system on the hard tissue (8,9). With this background in mind, the present study aimed to investigate the feasibility of co-registering OCT and CBCT images to enhance the accuracy of laser labeling and improve the intraoperative safety.

## Materials and Methods

**Fig 1:**
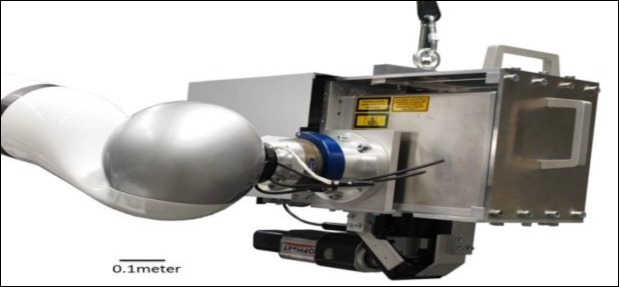
The combined system (designed by Hanover Laser center (LZH)) consisting of OCT and Er:YAG laser for simultaneous scanning and laser treatment attached to a robotic arm

For this project, a setup was selected combining a pulsed Er-YAG laser (λ=2940 nm, DPM 15 Laser Module, Pantec Engineering/ 3m.i.k.r.o.nTM) and an OCT (λ=930 nm, Thorlabs Ganymede Spectral Domain OCT) for the in situ observation and control of hard tissue ablation (Fig.1). 

The system was designed at Laser Zentrum Hannover (LZH, Hannover, Germany). The sample and cadaveric head scanning was accomplished using a Xoran xCAT ENT (Xoran Technologies LLC, Ann Arbor, Michigan, USA) with 120 kVp, 2.5 mA, and acquisition time of 40 sec, reconstruction field of view of 230×230×144 mm, voxel size of 0.3 mm; the isotropic data were exported to DICOM.

*Specimen preparation*

Three half head specimens of anonymous voluntary body donations were used without including any clinical or personal data. According to the European Union guideline on "Good Clinical Practice", the Declaration of Helsinki of the World Medical Association, and the Occupational Regulation for German Physicians (MBO-Ä 1997), vote of ethics committee is not necessary for such cases. 

**Fig 2:**
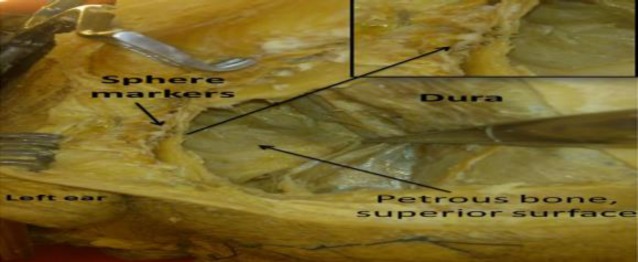
Spherical metal markers were placed on the lower edge of the craniotomy

First, a standard craniotomy for middle fossa approach above and mainly anterior to the external ear canal was performed in the three cadaveric specimens (Table.1). Afterwards, four spherical titanium markers were fixed to the lower edge of the craniotomy (Fig.2).

**Table 1:** Standard exposure in middle cranial fossa approach

1. Incision and Elevation of Skin Flaps (large reverse question mark from)
2. Elevate skin flap in plane of TP fascia (watch out for Frontal branch, In TP fascia proximally and deep to temporal fascia along zygomatic arch)
3. Muscle flap elevation from the calviarium using periosteal elevator
4. Creation of temporal craniotomy/bone flap (Approximately 4x5cm bone flap centered over zygoma)
5. Next, remove the bone flap
6. Freshen the edges of the craniotomy with the diamond burr
7. Retract dura and temporal lobe and place retractor
8. dissect the dura off of the middle fossa floor in a posterior to anterior direction (prevents avulsion of the GSPN, cauterize and cut middle meningeal artery)
9. Place the blade of retractor under the lip of the petrous ridge and engage in the retractor

The 1 mm-sized markers were positioned perpendicular to the laser beam in a non-linear arrangement at a distance of 1-2 mm side to side from each other. Thereafter, a CBCT scan was performed. The integrated OCT laser device and the skull were set up in their proper position during the data transfer to the software for segmentation and planning. The dura was retracted to ensure direct access to the superior surface of the temporal bone.

*Computer-assisted planning*

The IAC and the inner ear (superior semicircular canal and cochlea) were segmented in the CBCT data using MITK Workbench (Heidelberg, Germany). Custom software was used for the visualization of CBCT data, segmentation of structures, and placement of target points with the aim of marking the edge of the IAC. Therefore, the target points were placed on the bone surface directly above the IAC edge in the surgeon’s line of sight in a segmented three-dimensional image.

*Cone beam computed tomography-guided laser ablation*

**Fig 3:**
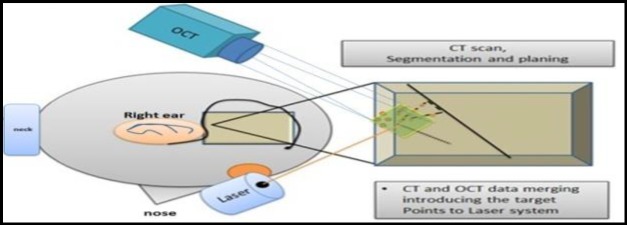
Schematic overview of the setup, 1) Specimen and MCF approach preparation, 2) CT scan, segmentation and planning, 3) OCT scan of markers 4) Acquisition of laser on the target points. 5) Laser ablation of target points. Magnified surgical view (right), laser (left bottom) and OCT view (left top)

The OCT-guided laser setup was used to track the titanium markers on the craniotomy edge in the lowest part of its cubic spectral field. The rest of this spectral field remained free for the pathway of the laser beam. The custom MATLAB (Massachusetts, USA) script was used to detect the spherical markers in the OCT image and transfer the planned target points with respect to the position of the spheres. The laser beam was directed accordingly; therefore, the laser was set with a power of 15 W, pulse frequency of 500 Hz, and pulse duration of 50-200 μs. Fine water was sprayed to cool the ablation area. The schematic overview of the strategy is shown in Figure 3 and summarized in Table 2. Finally, the accuracy was checked by placing the spheres on the laser-ablated sites and after CBCT scanning. Additionally, the exact positions of the desired and applied points were compared in the software.

**Table 2:** Major steps of the study

Preparation of explanted human cadaver head
Marking the surface with four spherical titanium markers
CBCT scan of samples
Planning in software
Segmentation of the IAC and Inner ear structures (MITK Workbench)
Definition of target points (TP) on scanned image (custom software)
Image acquisition
Surface scan by OCT, finding the spherical markers
Acquisition of spheres parameter in CBCT and OCT images (MATLAB)
Finding of the TP with the laser system software (MATLAB)
Acquisition of laser view and OCT image using TP and the titanium markers, respectively
Laser ablation based on the all complex data to TP, caring the segmented area

## Results

**Fig 4:**
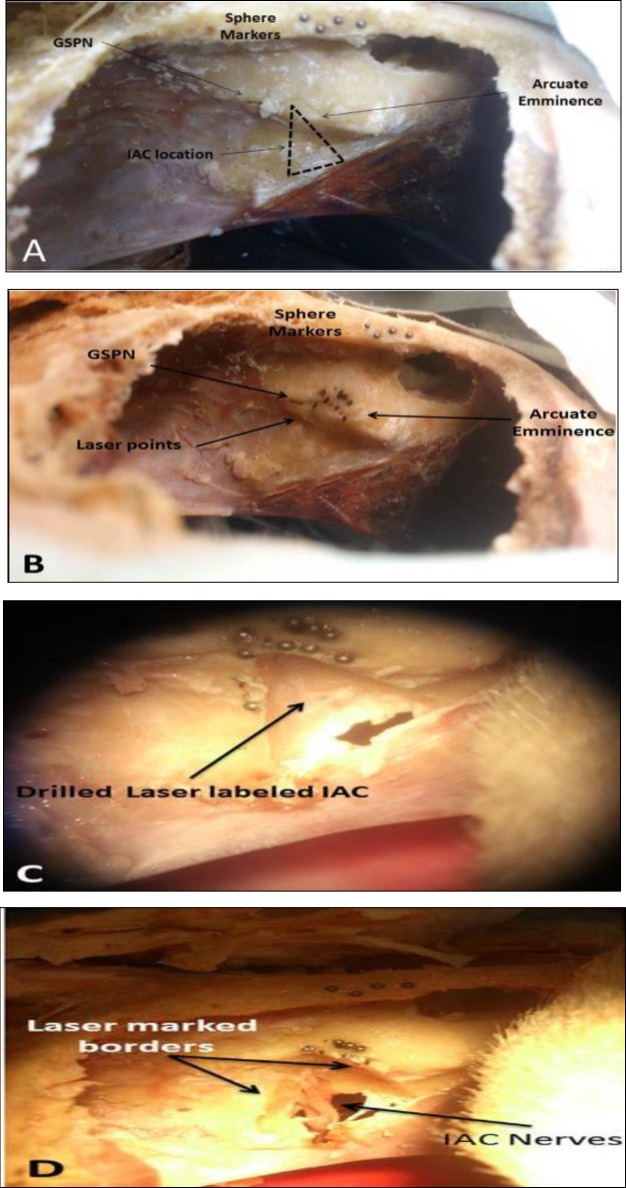
A: surgical view, B: Laser ablated site in V form for IAC and straight points for SSCC, (Brain was removed after ablation), C: Marking the laser ablated points and drilling the bone over the IAC, D: exposing the IAC contents

The preparation of the cadaver specimens was easier than that of a real person in an operation due to the access to an avascular field, and no fear of injuring vital organs, such as the brain. However, cadaveric brain retraction is harder; as a result, the preparation took about 45 min. Spatial overlap between the OCT imaging range and the addressable focus positions of cutting laser comprises a volume of 10×10×10 mm (3). Therefore, the artificial markers had to be placed close to the target area. Consequently, the titanium markers were configured within the lower part of the spectral view. The IAC border marked in the software was ablated with a laser in a V-shape (Fig.4).

Most of the heat energy was absorbed by the ablated bone; therefore, no sign of charring in the ablation area was observed as long as the bone was kept wet. However, laser ablation points were more visible using a higher energy and dryness or defocus ablation. Regarding the selected laser setting, the system was applicable for a gentle processing of the biological hard tissue with minor thermal damage. With each ablation cycle, the bone ablated around 300 μm in depth. The actual speed of the laser focus spot was 4 mm/s. With regard to the time, placing the markers on the craniotomy edge took 5 min, and the intraoperative imaging (CBCT) lasted 15 min. Furthermore, the segmentation and the planning of the target points took 10-15 min. The setting of the laser-OCT integrated system on the head in the proper position and OCT scans took around 10 min, and laser ablation was generally in second. The total process was carried out in 30-35 min.

In the study setup, better laser-OCT access and action range were obtained by tilting the head until the petrous surface was placed in an angle of about 45-90° to the beam. The parametric position of marked points, which were marked with spheres, in post-procedure scan data and previous data were analyzed using the Excel software(ver.15), showing a mean error of 300 μm. Subsequently, the head of the project (O.M) dissected the specimens and approved the accuracy as well.

## Discussion

It is not easy for the surgeons to expose the IAC in the middle fossa approach due to the anatomic variation of the temporal bone, different degrees of the pneumatization of the temporal bone, and the correct identification of the landmarks (10). The preservation of the neighboring anatomical structures (i.e., superior semicircular canal, cochlea, and facial nerve) is vital for the safety and positive outcome of the procedure. Our experimental results showed the applicability of real-time optical navigation in one of the most difficult and complex surgical approaches.

The OCT as an imaging technology is used for different otologic goals, for example cochlea visualization and implantation sites (11-13). Zhang et al. used OCT to accurately perform cochleostomy (14). This technique is also an attractive candidate for compact, non-invasive, and real-time imaging of laser irradiated material. Some researchers have introduced and combined OCT with surgical lasers for qualitative and quantitative monitoring of the laser ablation process in various soft and hard tissues (15,16)_. _

Goel et al. employed the OCT for synergy between imaging and surgery, which improved the operative outcomes and reduced patient morbidity (17). Eilers et al. presented the first results of an automatic segmentation of OCT images and a multi-modal image registration based on the mutual information. They utilized the CBCT imaging system for scanning, but in order to increase the accuracy, they added a high-resolution OCT to overcome the CBCT image resolution. Their study was designed to perform a minimal invasive surgery as a part of robotic-assisted surgery for cochlear implantation (18).

Due to multiple advantages, such as contact-free processing, arbitrary cutting geometries, and high precision, the laser could be very helpful in image-guided laser surgery (19,20). In a minimally invasive cochlear implantation, Eilers et al. integrated the IGS into a robotic surgery (21). Caversaccio et al. discussed the applicability and advantages of navigation and robotics of the lateral skull base (22). Robots are able to handle more complex data; therefore, it is possible to incorporate computed tomography (CT) image data, OCT scan, and laser in robots and establish a new strategy to manage the disease.

The xCAT ENT scanner is a volume CT scanner that gives an effective radiation dose of 0.17 mSv, compared with 0.07 mSv for a conventional skull X-ray and 2 mSv for a standard head CT. This radiation risk seems small, compared to the potential advantages in conditions where normally intra-operative imaging would be considered (23,24).

Recently, some research groups have been using OCT to guide laser ablation (14,25). The first group published their study about hard tissue ablation using laser and OCT guidance (25), while the second group focused on precise cochleostomy in porcine with a CO_2_ laser under OCT control (14).

To the best of our knowledge, this study is the first attempt to merge three different techniques, namely CT scan, OCT image, and laser beam, in order to simulate a robotic setup for image-guided surgery in the bony structures of the human lateral skull base. One of the most encouraging results of the present was the surgical duration, with the precise setup taken about 30 min. In the standard approach, the blue lining of the superior semicircular canal and finding the exact position of IAC are critical and time-consuming. The safe blue lining of the canal requires sufficient experience. This computational method was well defined; moreover, it did not require the blue linin, thereby saving time. In addition, in case of lacking adequate experience, this technique avoids the risk of inner ear damage. Marking the border was helpful for the safe and faster drilling of IAC roof because the surgeon was sure about the exact anatomy of the neighboring structures.

The main limitation was the OCT working distance. The effective working distance was 60 mm, adjusted to 100 mm; therefore, with large distances, the application was restricted. The main part of the IAC is the lateral part adjacent to the cochlea, canal, and nerve. Accordingly, the marking of the lateral part, mainly included in the working distance, is enough to overcome this limitation.

This technique is also limited to imaging 2-3 mm below the surface in biological tissues and 1 mm in bony structures; furthermore, it provides a good resolution in a lateral spectral view of approximately 1 cm^2^. The field of view is sufficient for navigating the laser beam (25,26). The OCT and Er:YAG laser beams were combined by a dichroic mirror. A 3D scanner system (i.e., 2D scanner and dynamic focusing unit), in combination with a focusing lens, creates a maximum ablation volume of 10×10×10 mm (3,12). In this volume, visibility and laser calibration is accurately guaranteed. 

Setting up the novel laser-adapted OCT system through this approach could open the way for its use in different surgical approaches on the lateral skull base. The bone ablated depth was around 300 μm. The actual structures were deeper, and consequently safer from damage. This issue is very important to preserve the vital neighborhood structures.

The accuracy was shown in the analyses of data, dissection of ablation points, and IAC location. The overall accuracy was 300 μm according to target and ablated points. This is a great achievement in the lateral skull base; however, we believe that it can be improved with better CBCT and OCT image resolution. No limitations were found to exclude any cases for this technology. Future research will focus on minimization of the computational time, data transferring, implementation of adaptive algorithms to automate parameterization, and design of a well-adapted tracking method for OCT registration.

## Conclusion

The present study demonstrated the feasibility of interactive guidance and marking of the surgical site during laser tissue ablation. Merging the different imaging technologies, namely CBCT and OCT, for a highly precise navigation in the lateral skull base, along with the laser ablation ability, is an innovative method. 
